# MEYE: Web App for Translational and Real-Time Pupillometry

**DOI:** 10.1523/ENEURO.0122-21.2021

**Published:** 2021-09-30

**Authors:** Raffaele Mazziotti, Fabio Carrara, Aurelia Viglione, Leonardo Lupori, Luca Lo Verde, Alessandro Benedetto, Giulia Ricci, Giulia Sagona, Giuseppe Amato, Tommaso Pizzorusso

**Affiliations:** 1 Department of Neuroscience, Psychology, Drug Research and Child Health (NEUROFARBA), University of Florence, 50135 Florence, Italy; 2 Institute of Neuroscience, National Research Council, 1-56124 Pisa, Italy; 3 Department of Developmental Neuroscience, IRCCS Stella Maris Foundation, 56128 Pisa, Italy; 4 Laboratory of Biology BIO@SNS, Scuola Normale Superiore, 1-56124 Pisa, Italy; 5 Istituto di Scienza e Tecnologia dell’Informazione (ISTI), 1-56124 Pisa, Italy; 6 Department of Translational Research on New Technologies in Medicine and Surgery, University of Pisa, 56127 Pisa, Italy

**Keywords:** arousal, neural network, oddball, pupillometry, virtual reality, web app

## Abstract

Pupil dynamics alterations have been found in patients affected by a variety of neuropsychiatric conditions, including autism. Studies in mouse models have used pupillometry for phenotypic assessment and as a proxy for arousal. Both in mice and humans, pupillometry is noninvasive and allows for longitudinal experiments supporting temporal specificity; however, its measure requires dedicated setups. Here, we introduce a convolutional neural network that performs online pupillometry in both mice and humans in a web app format. This solution dramatically simplifies the usage of the tool for the nonspecialist and nontechnical operators. Because a modern web browser is the only software requirement, this choice is of great interest given its easy deployment and setup time reduction. The tested model performances indicate that the tool is sensitive enough to detect both locomotor-induced and stimulus-evoked pupillary changes, and its output is comparable to state-of-the-art commercial devices.

## Significance Statement

Alteration of pupil dynamics is an important biomarker that can be measured noninvasively and across different species. Although pupil size is driven primarily by light, it can also monitor arousal states and cognitive processes. Here we show an open-source web app that, through deep learning, can perform real-time pupil size measurements in both humans and mice, with accuracy similar to commercial-grade eye trackers. The tool requires no installation, and pupil images can be captured using infrared webcams, opening the possibility of performing pupillometry widely, cost-effectively, and in a high-throughput manner.

## Introduction

Pupillometry, the measurement of pupil size fluctuations over time, provides useful insights into clinical settings and basic research activity. Light level is the primary determinant of pupil size, although non-light-driven pupil fluctuations, widely assumed as an indicator of arousal through locus coeruleus activity, can be used to index brain state across species (McGinley et al., 2015; Lee and Margolis, 2016; Reimer et al., 2016). Higher cognitive and emotional processes are also able to evoke tonic or phasic pupillary changes, such as attention (Binda et al., 2013a), memory load (Wierda et al., 2012), novelty (Angulo-Chavira et al., 2017; Krebs et al., 2018; Montes-Lourido et al., 2021), pain (Connelly et al., 2014; Azevedo-Santos and DeSantana, 2018; Charier et al., 2019), and more general cortical sensory processing (Binda et al., 2013b; Lee and Margolis, 2016) in humans and in animal models.

A growing body of work shows how pupillometry can be used as a possible biomarker for numerous neurologic and psychiatric conditions in early development and adult subjects (Aleman et al., 2004; Blaser et al., 2014; Rorick-Kehn et al., 2014; Frost et al., 2017; Nyström et al., 2018; Chougule et al., 2019; Gajardo et al., 2019; Oh et al., 2019, 2020; Artoni et al., 2020; Burley and van Goozen, 2020; Iadanza et al., 2020; Obinata et al., 2020; Winston et al., 2020; El Ahmadieh et al., 2021). Spontaneous and voluntary modulation of pupil fluctuations has also been used to facilitate human–computer interaction in normal subjects (Mathôt et al., 2016; Beggiato et al., 2018; Ponzio et al., 2019) and patients with severe motor disabilities. For example, pupil dynamics is used to assess communication capability in locked-in syndrome, a crucial factor for the determination of a minimally conscious state (Olivia et al., 2013; Stoll et al., 2013). Pupillometry is also becoming a valuable tool for child neurology, to facilitate risk assessment in infants. For example, the pupil light reflex (PLR) during infancy seems to predict the later diagnosis and severity of autism spectrum disorders (ASDs; Nyström et al., 2018). Intriguingly, pupil alterations are also present in several ASD mouse models (Artoni et al., 2020).

Pupillometry has several advantages compared with other physiological methods: it is noninvasive and can be performed by nonspecialized personnel on noncollaborative and preverbal subjects (like infants), allowing the design of longitudinal experiments to permit temporal specificity. More importantly, it can be conducted similarly across different species from mice to humans, guaranteeing maximal translatability of the protocols and results (Aleman et al., 2004; Rorick-Kehn et al., 2014; Artoni et al., 2020). Given these assumptions, it is vital to introduce a simple, versatile tool used in a range of settings, from the laboratory to the clinical or even domestic environment. Available open-source methods require complicated steps for the installation and configuration of custom software not suitable for nontechnical operators. Moreover, these tools were tested exclusively in one species [mice (Privitera et al., 2020), humans (Yiu et al., 2019)], and none of them were applied in cognitive experiments that usually involve small pupil changes associated with high variability.

In this work, we have developed a deep learning tool called MEYE, using convolutional neural networks (CNNs) to detect and measure real-time changes in pupil size both in humans and mice in different experimental conditions. Furthermore, the MEYE web app, performs pupil area quantification and blink detection, all within a single network. By embedding artificial intelligence algorithms in a web browser to process real-time webcam streams or videos of the eye, MEYE can be used by nontechnical operators, opening the possibility to perform pupillometry widely, cost-effectively, and in a high-throughput manner. This architecture is resistant to different illumination conditions, allowing the design of basic neuroscience experiments in various experimental settings, such as behavior coupled with electrophysiology or imaging such as two-photon microscopy. To describe the performance of the MEYE web app in different settings, we tested the app in both mice and humans. In mice, we recorded both running speed and pupil size during visual and auditory stimulation (AS). In humans, we tested MEYE capabilities to detect the PLR. Furthermore, we performed a visual oddball paradigm (Liao et al., 2016; Aggius-Vella et al., 2020; LoTemplio et al., 2021), comparing pupil size and eye position measurements obtained from MEYE with one of the most used commercial eye-tracker systems: the EyeLink 1000. Finally, we released a dataset of 11,897 eye images that can be used to train other artificial intelligence tools.

## Materials and Methods

### Datasets

**Figure 1.**
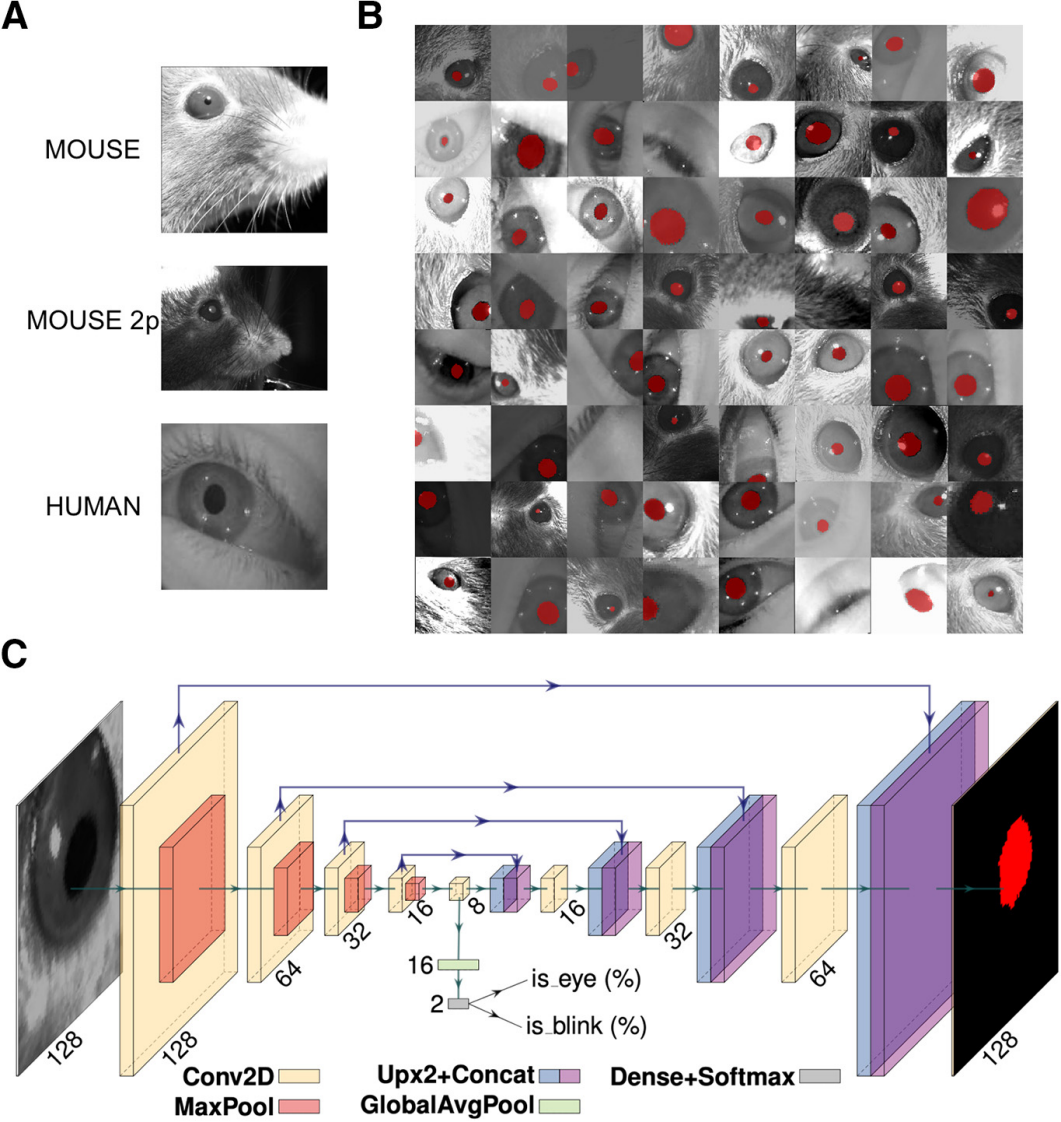
Dataset, CNN architecture, and performances. ***A***, Examples of images taken from the dataset. The first image depicts a head-fixed mouse with dark pupils, the second one is a head-fixed mouse with a bright pupil, during two-photon microscope sessions. The last image is a human eye taken during experiments wearing virtual reality goggles. ***B***, The 64 examples of data augmentation fed to CNN. The images are randomly rotated, cropped, flipped (horizontally or vertically), and changed in brightness/contrast/sharpness. ***C***, CNN architecture with an encoder–decoder “hourglass” shape. The encoder part comprises a sequence of convolutional layers. Starting from the last encoder output, the decoder part iteratively upsamples and fuses feature maps with corresponding encoder maps to produce the output pixel map. The pixel probability map and eye/blink probabilities are computed by applying the sigmoid activation to the network outputs in an element-wise manner.

For this study, we collected a dataset (Fig. 1*A*) composed of 11,897 grayscale images of human (4285) and mouse (7612) eyes. The majority of the pictures are of mouse eyes during head fixation sessions (5061 sessions) in a dark environment using infrared (IR; 850 nm) light sources. In this environment, the pupil is darker than the rest of the image. We also collected mouse eyes [two-photon imaging mice (2P mice), 2551] during two-photon Ca^2+^ imaging. In this particular condition, the pupil is inverted in color and tends to be brighter than the iris. Finally, we acquired images of human eyes in IR light (4285 eyes) during virtual reality (VR) experiments (wearing a headset for virtual reality), using an endoscopic camera (www.misumi.com.tw/). The dataset contains 1596 eye blinks, 841 images in the mouse, and 755 photographs in the human datasets. Five human raters segmented the pupil in all pictures (one per image), using custom labeling scripts implemented in MATLAB or Python by manual placement of an ellipse or polygon over the pupil area. Raters flagged blinks using the same code.

### CNN architecture

The CNN model (Fig. 1*C*) takes 128 × 128 grayscale images as input and produces the following three outputs: (1) a 128 × 128 probability map of each pixel belonging to the pupil; (2) the probability the image contains an eye; and (3) the probability the image depicts a blinking eye. We evaluated three architectures: two were based on DeepLabv3+ (Chen et al., 2018), a family of image segmentation models that use atrous convolutions and spatial pyramid pooling, which are known to improve robustness to scale changes. The two models differ for the CNN backbone adopted, respectively, ResNet-50 (He et al., 2016) and MobileNet V3 (Howard et al., 2019) CNNs. The third evaluated model is a specialized variant of the U-Net architecture (Ronneberger et al., 2015), a widely used CNN in image segmentation tasks. The model has an encoder–decoder “hourglass” architecture; the encoder part comprises a sequence of convolutional layers with ReLU activation and 2 × 2 maximum pooling operation, each halving the spatial resolution of feature maps at every layer; this produces a sequence of feature maps of diminishing spatial dimensions that provides both spatially local information and global context for the subsequent steps. Starting from the last encoder output, the decoder part iteratively upsamples and fuses feature maps with corresponding encoder maps, using convolutional layers, to produce the output pixel map. All convolutional layers have 16 3 × 3 kernels and pad their input to obtain output of the same shape. Convolutional layer upsampling and downsampling were changed by a factor of 2 (Fig. 1*C*). In all the tested architectures, eye and blink probabilities are predicted by an additional branch that applies global average pooling and a two-output fully connected layer to the bottleneck feature map. The pixel probability map and eye/blink probabilities are computed by applying the sigmoid activation to the network outputs element wise. Among the tested architectures, we chose to adopt the UNet variant in this work, as we observed it provided the best tradeoff in terms of speed and segmentation quality (for further information see: https://github.com/fabiocarrara/meye/wiki/MEYE-Models).

### Augmentation, training, and validation

We randomly split the dataset into training, validation, and test subsets following a 70%/20%/10% split. We performed strong data augmentation during the training phase by applying random rotation, random cropping, random horizontal and vertical flipping, and random brightness/contrast/sharpness changes; images were resized to 128 × 128 before feeding them to the CNN (Fig. 1*B*).

For validation and test images, we used a 128 × 128 crop centered on the pupil. We computed the binary cross-entropy for all outputs (pixels and eye/blink logits) and took the sum as the loss function to minimize. The network was trained with the AdaBelief optimizer (Zhuang et al., 2020) for 750 epochs with a learning rate of 0.001. The best performing snapshot on the validation set was selected and evaluated on the test set.

### MEYE: web browser tool

We built a web app for pupillometry on recorded or live-captured videos harnessing a CNN segmentation model as the core component. The trained models have been converted to a web-friendly format using *TensorFlow.js*, thus enabling predictions on the user machine using a web browser.

This choice greatly facilitates the deployment and reduces setup time, as a modern web browser is the only minimum requirement. Once loaded, an Internet connection is not mandatory, as no data leaves the user’s browser, and all the processing is performed on the user’s machine. This implies that performance greatly depends on the user’s hardware; if available, hardware (graphics processing unit (GPU)] acceleration is exploited automatically by *TensorFlow.js*. In our tests, a modern laptop shipping an Intel(R) Core(TM) i7-9750H 2.60 GHz CPU and an Intel(R) UHD Graphics 630 GPU can process up to 28 frames/s (fps).

The web app also offers additional features that facilitate the recording process, such as the following: processing of prerecorded videos or real-time video streams captured via webcam; ROI placement via user-friendly web user interface (UI; drag and drop) and automatic repositioning following tracked pupil center; embedded tunable preprocessing (image contrast/brightness/gamma adjustment and color inversion) and postprocessing (map thresholding and refinement via mathematical morphology); support for registering trigger events; live plotting of pupil area and blink probability; and data export in CSV format including pupil area, blink probability, eye position, and trigger channels.

### Behavioral experiments on mice

#### Animal handling

Mice were housed in a controlled environment at 22°C with a standard 12 h light/dark cycle. During the light phase, a constant illumination <40 lux from fluorescent lamps was maintained. Food (standard diet, 4RF25 GLP Certificate, Mucedola) and water were available *ad libitum* and were changed weekly. Open-top cages (36.5 × 20.7 × 14 cm; 26.7 × 20.7 × 14 cm for up to five adult mice; or 42.5 × 26.6 × 15.5 cm for up to eight adult mice) with wooden dust-free bedding were used. All the experiments were conducted following the directives of the European Community Council and approved by the Italian Ministry of Health (1225/2020-PR). All necessary efforts were made to minimize both stress and the number of animals used. The subjects used in this work were three female 3-month-old C57BL/6J mice for the auditory stimulation and five male 2-month-old mice for the VR experiment.

#### Surgery

The mouse was deeply anesthetized using isoflurane (3% induction, 1.5% maintenance). Then it was mounted on a stereotaxic frame through the use of ear bars. Prilocaine was used as a local anesthetic for the acoustic meatus. The eyes were treated with a dexamethasone-based ophthalmic ointment (Tobradex, Alcon Novartis) to prevent cataract formation and keep the cornea moist. Body temperature was maintained at 37°C using a heating pad monitored by a rectal probe. Respiration rate and response to toe pinch were checked periodically to maintain an optimal level of anesthesia. Subcutaneous injection of lidocaine (2%) was performed before scalp removal. The skull surface was carefully cleaned and dried, and a thin layer of cyanoacrylate was poured over the exposed skull to attach a custom-made head post that was composed of a 3D-printed base equipped with a glued set screw (12 mm long, M4 thread; catalog #SS4MS12, Thorlabs). The implant was secured to the skull using cyanoacrylate and UV curing dental cement (Fill Dent, Bludental). At the end of the surgical procedure, the mice recovered in a heated cage. After 1 h, mice were returned to their home cage. Paracetamol was used in the water as antalgic therapy for 3 d. We waited 7 d before performing head-fixed pupillometry to provide sufficient time for the animal to recover.

#### Head fixation

In the awake mouse head fixation experiments, we used a modified version of the apparatus proposed by Silasi et al. (2016), equipped with a 3D-printed circular treadmill (diameter, 18 cm). Components are listed in Table 1. A locking ball socket mount (TRB1/M) was secured to an aluminum breadboard (MB2020/M) using two optical posts (TR150/M-P5) and a right-angle clamp (RA90/M-P5). The circular treadmill was blocked between the base plate pillar rod and the optical post through a ball-bearing element (BU4041, BESYZY) to allow the spinning of the disk with low effort. To couple the head-fixing thread on the mouse to the locking ball, an ER025 post was modified by retapping one end of it with M4 threads to fit the ball and socket mount. Velocity was detected using an optical mouse under the circular treadmill. Pupillometry was performed using a USB camera (oCam-5CRO-U, Withrobot) equipped with a 25 mm M12 lens connected to a Jetson AGX Xavier Developer Kit (NVIDIA) running a custom Python3 script (30 fps). The Jetson hardware was connected with an Arduino UNO microcontroller board through GPIO (general purpose input/output) digital connection. The Arduino UNO managed the auditory stimuli through a speaker (3 inch; model W3-1364SA, Tang Band Speaker).

**Table 1:** Head fixation apparatus components (thorlabs.com)

Part number	Description	Quantity	Price (€)
TRB1/M	Locking ball and socket mount M4	1	55.83
TR150/M-P5	Optical post M4–M6, 150 mm, 5 pack	1	29.97
RA90/M-P5	Right-angle clamp	1	45.7
MB2020/M	Aluminum breadboard	1	72.3
RS075P/M	Pedestal pillar post	1	21.63
SS4MS12	Set screws 12 mm long, M4	1	5.61
AP4M3M	Adaptor M4-M3	5	1.91
ER025	Cage assembly rod	5	4.73
SS6MS12	Set screws 12 mm long, M6	1	5.55
CF038C-P5	Clamping fork	1	46.49
Total			289.72

#### Behavioral procedures

Mice were handled for 5 min each day during the week preceding the experiments; then, they were introduced gradually to head fixation for an increasing amount of time for 5 d. During days 1 and 2, we performed two sessions of 10 min of head fixation, one in the morning and one in the afternoon. On day 3, we performed one session of 20 min; on day 4, 30 min; and on day 5, 35 min. Each recording started with 5 min of habituation. We exposed the animal to auditory stimuli during the last day. During each head fixation session, a curved monitor (24 inches; model CF390, Samsung) was placed in front of the animal (distance, 13 cm) showing a uniform gray with a mean luminance of 8.5 cd/m^2^. The frequency of tone 1 was 3000 Hz, and of tone 2, 4000 Hz, both at 70 dB, a duration of 20 s, and an interstimulus interval of 120 s. Virtual reality was composed of a γ-linearized procedural virtual corridor with episodic visual stimulation written in C# and Unity. The virtual corridor was composed of sine-wave gratings at different orientations (wall at 0°; floor at 90°), and spatial frequencies (from 0.06 to 0.1 cycles/°). The position of the animal in the virtual corridor was updated using an optical mouse connected to the circular treadmill. The episodic visual stimulus consisted of a square wave grating patch of 55° (in width and height) of visual space in the binocular portion of the visual field. The grating parameters were as follows: luminance, 8.5 cd/m^2^; orientation, 0°; contrast, 90%; spatial frequency, 0.1 cycles/°; drifting, 0.5 cycle/s.

#### Data analysis

Data has been analyzed using Python 3. All tracks were loaded, and blink removal was applied using the blink detector embedded in MEYE. Blink epochs were filled using linear interpolation and median filtering (0.5 s). Spearman ρ rank-order correlation was performed using the function *corr* from Python library *pingouin* (Vallat, 2018). The *z*-score was obtained for each trial using the formula $z=\left(x-{\bar{x}}_{\text{baseline}}\right)/s_{\text{baseline}}$, where and ${\bar{x}}_{\text{baseline}}$ and $s_{\text{baseline}}$ were respectively the average and the SD of the baseline. To evaluate whether event-related transients (ERTs) amplitude was significantly different from baseline, a two-way repeated-measures ANOVA was computed on each time sample using the *pingouin* function *rm_anova. Post hoc* analyses and multiple-comparison *p* value correction were conducted using the function *pairwise_t tests* from *pingouin*. For both pupil size and velocity, we compared each time sample after sensory stimulation with the average value of the baseline, adjusting the *p* values using Benjamini–Hochberg FDR correction. For the behavioral state analysis, locomotion activity was identified using a threshold algorithm. We tagged as moving all the samples in which velocity was ≥10% with respect to the maximal speed of the animal. Paired *t* tests between behavioral states were performed using the *function t* test from *pingouin*. Comparison of eyes movements was conducted normalizing (range between −1 and 1) data from both setups, upsampling MEYE data from 15 to 1000 fps using linear interpolation, and then calculating the mean absolute error (MAE), which was performed using the Python function *mean_absolute_error* from the library *sklearn*.

### Behavioral experiments on humans

#### PLR

Pupillometry has been performed using a MacBook Pro (Retina, 13 inch, Early 2015, Intel Core i5 dual-core 2.7 GHz, 8GB of RAM, Intel Iris Graphics 6100 card, 1536 MB) running the MEYE application on Firefox (84.0). The tool is able to compute online pupil size quantification, plotting the instantaneous pupil area and saving the results on file. Furthermore, the tool accepts four independent manual push-button triggers (keys “T” or “Y” on the keyboard). This feature allowed us to annotate stimulation events. A USB IR webcam (model Walfront5k3psmv97x, Walfront) equipped with a Varifocal 6–22 mm M12 objective (catalog #149129, Sodial) was used to acquire images of the eye. The camera was equipped with six IR LEDs to illuminate the eye uniformly, optimizing contrast between the iris and the pupil. Photic stimulation was delivered using an Arduino Due (Arduino) microcontroller connected via USB to the notebook and programmed to emulate a keyboard. The Arduino emulates a keyboard (using the *keyboard. h* library) to send event triggers to MEYE in the form of keystroke events. The microcontroller drives a stripe of four LEDs (WS2813, WorldSemi) using the *FastLED. h* library, flashing bright white light for 500 ms with an interstimulus interval of 5 s (see Fig. 3*A*). The subject sat in front of a monitor screen (24 inches; model CF390, Samsung) at a distance of 60 cm, with the head stabilized by a chin rest and instructed to maintain fixation on a small dot presented in the center of the screen for the whole duration of the recording (57 s). A total of 10 flash stimuli have been presented through the strip of LEDs mounted above the screen.

#### Oddball paradigm corecordings

To compare the performances shown by the CNN system with that of a state-of-the-art commercial software, we coregistered pupillometry using MEYE and an EyeLink 1000 system, while nine participants (three males, six females; average age, 28.78 years) executed an oddball paradigm. The experiment was conducted in a quiet, dark room. The participant sat in front of a monitor screen (88 × 50 cm) at a distance of 100 cm, with their head stabilized by a chin rest. The viewing was binocular. Stimuli were generated with the PsychoPhysics Toolbox routines (Brainard, 1997; Pelli, 1997) for MATLAB (MATLAB r2010a, MathWorks) and presented on a γ-calibrated PROPixx DLP LED projector (VPixx Technologies) with a resolution of 1920 × 1080 pixels, and a refresh rate of 120 Hz. Pupil diameter was monitored at 1 kHz with an EyeLink 1000 system (SR Research) with an infrared camera mounted below the screen and recording from the right eye. The participant was instructed to maintain fixation on a small dot (0.5°) presented in the center of the screen for the whole duration of the recording (300 s). In this study, the visual stimuli consisted of the appearance of a high-probability stimulus (80% of times) defined as “Standard” and a lower probability stimulus (20% of times) defined as “Target.” The Standard stimulus consisted of a 100% contrast-modulated annular grating (mean luminance, 25 cd/m^2^), horizontally oriented, with a spatial frequency of 0.5 cpd, and with inner and outer diameters of 1.5° and 5°, respectively. The edges of the annulus were smoothed by convolving the stimulus with a Gaussian mask (σ = 0.5°). The Target stimulus had the same parameters as the Standard stimulus except that the orientation that was 45° (see Fig. 4*A*). The presentation duration of each trial, either the Standard (0°) or Target (45°) trial, was 200 ms with an intertrial interval between two consecutive trials of 2800 ms. The phases of both the Target and the Standard stimuli were randomized across trials. The participants were instructed to press a button for a Target stimulus and not to respond for a Standard stimulus. The *z*-scored ERTs were computed as described for mice. The correlation was performed by taking the amplitude of the peaks of the Targets for each subject using both EyeLink and MEYE, and then performing the Spearman ρ rank-order correlation between the two measures.

#### Eye movement corecordings

For eye-tracking recording, we used both the MEYE tool and EyeLink 1000, as described above. In the smooth pursuit condition, a small dot (0.5°) moved on the screen horizontally, changing direction every 20° of the visual field with a constant velocity of 8°/s. In the saccades condition, every 2.5 s the small dot abruptly changes position horizontally with a span of 20°.

#### Offline video analysis

The MP4 videos were loaded into MEYE, and the parameters were chosen by visually inspecting the quality of the pupillometry. Threshold values were 0.25, 0.15, and 0.5, with morphology FALSE, TRUE, TRUE for “human,” “mouse,” and “2P-mouse” videos respectively. Once the video analysis was completed, the CSV file was loaded into Python. Blink removal and linear interpolation were applied, then the track was plotted using the Python library *matplotlib*.

### Data availability

The code and web app are freely available on Github: github.com/fabiocarrara/meye. MEYE is available at: www.pupillometry.it. MEYE wiki is available at: https://github.com/fabiocarrara/meye/wiki. The dataset is available on: https://doi.org/10.5281/zenodo.4488164.

## Results

### Pupillometry in head-fixed mice

**Figure 2.**
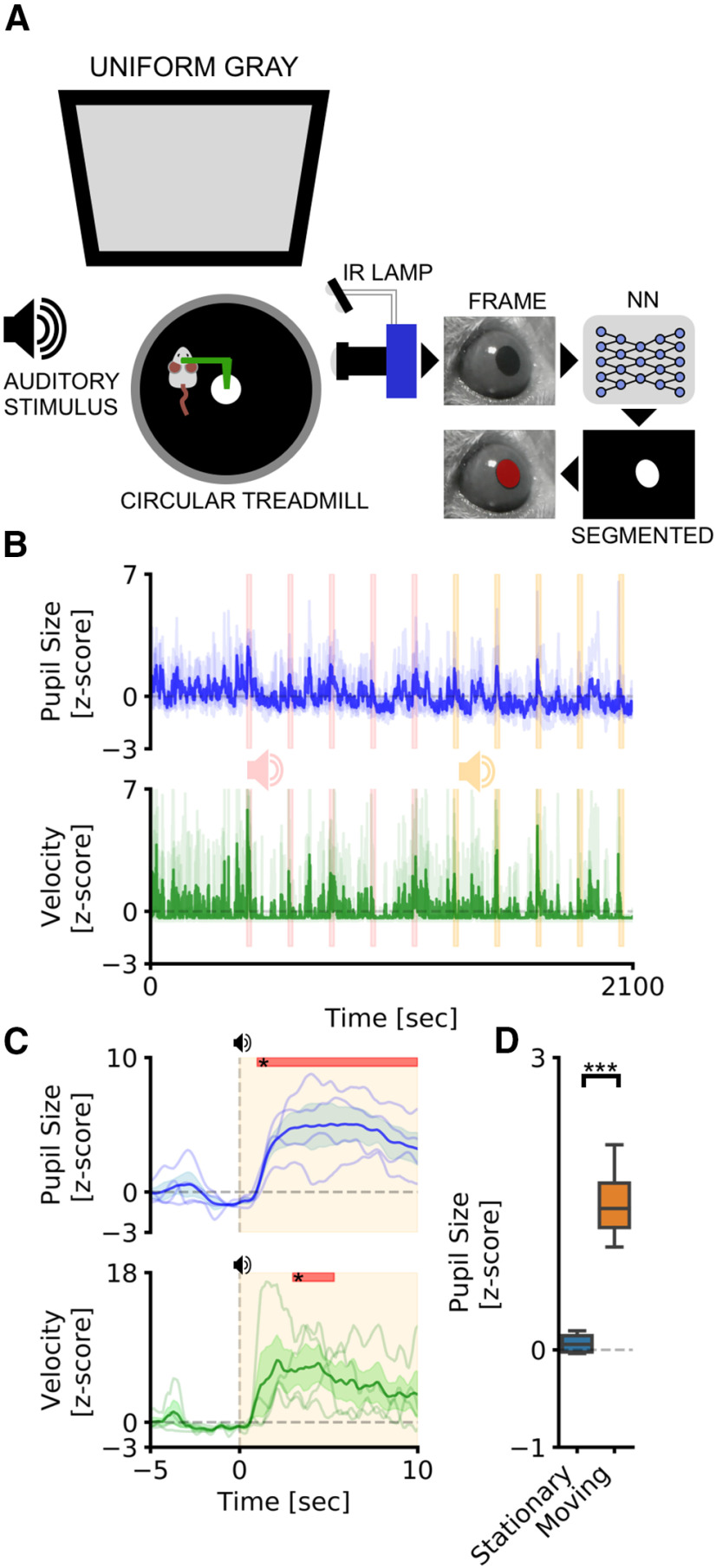
Pupillometry in head-fixed mice. ***A***, Setup for head-fixed pupillometry in the awake mouse. The head of the mouse is fixed to a custom-made metal arm equipped with a 3D-printed circular treadmill to monitor running behavior. In the meantime, pupillometry is performed using CNN. ***B***, The average fluctuations of pupillometry and velocity in all experimental mice. Dashed pink and yellow areas represent the onset and duration of auditory stimuli. Evoked peaks in both pupil size (blue line) and velocity (green line) are clearly noticeable during auditory stimulation. ***C***, Average event-related transients for both pupil size and velocity. Colored areas represent stimulus onset and duration. Red areas in the top part of the plot represent statistically significant data points. ***D***, Sensibility of the system to detect locomotor-induced arousal fluctuations. Average pupil size is significantly affected by the behavioral states of the animal. During running epochs (Moving) the pupil is significantly more dilated than during the resting state (Stationary). ***p > 0.001.

**Figure 3.**
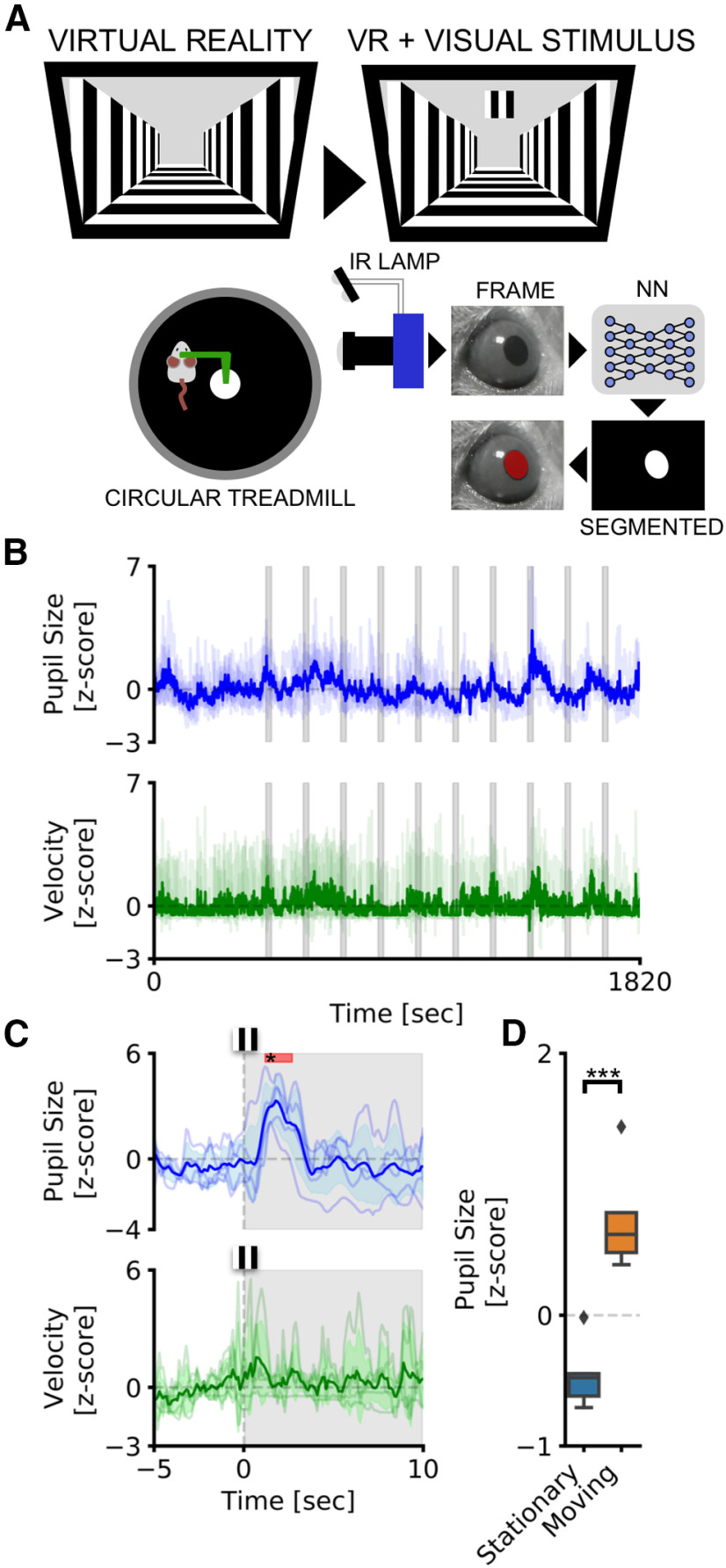
Pupillometry and VR in head-fixed mice. ***A***, Setup for head-fixed pupillometry in VR in the awake mouse, showing the habituation phase (left), in which only the virtual corridor is shown, and the stimulation phase, in which a visual stimulus appears above the virtual corridor, in the binocular portion of the visual field. ***B***, The average fluctuations of pupillometry and velocity in all experimental mice. Dashed gray areas represent the onset and duration of auditory stimuli. ***C***, Average event-related transients for both pupil size and velocity. Colored areas represent stimulus onset and duration. Red areas in the top part of the plot represent statistically significant data points. ***D***, Sensibility of the system to detect locomotor-induced arousal fluctuations. Average pupil size is significantly affected by the behavioral states of the animal. During running epochs (Moving), the pupil is significantly more dilated than during the resting state (Stationary). ***p > 0.001.

We tested our CNN-based pupillometer in two behavioral experiments involving locomotion-induced and stimulus-evoked pupillary changes. Pupil size was simultaneously recorded with running speed from head-fixed mice free to run on a circular treadmill (Fig. 2*A*). We used two different stimulation protocols: AS, and visual stimulation while the animal is freely exploring VR. The VR experiment included an initial period of habituation in which the animal navigated inside a virtual corridor for 5 min. After this period, a square visual stimulus was presented in the binocular portion of the visual field (duration, 20 s; interstimulus interval, 120 s, 10 times; Fig. 3*A*,*B*). The AS experiment was conducted with the same structure as the VR experiment and using auditory stimulus previously used to induce a defensive behavior detectable as a pupillary and behavioral response (Xiong et al., 2015; Wang et al., 2019; Hersman et al., 2020; Li et al., 2021). An initial period of habituation was followed by auditory stimulation using two tones (tone 1, 3 kHz; tone 2, 4 kHz; duration, 20 s; interstimulus interval, 120 s; Fig. 2*A*,*B*). We first set out to evaluate whether CNN can detect event-related pupil transients (i.e., ERTs) because of sensory stimulation in the VR. We averaged pupil size and running velocity during a 15 s temporal window centered on visual stimulation (Fig. 3*C*). We detected a significant pupillary dilation after the onset of the visual stimulus and no changes in evoked locomotion (pupil: *p* < 0.001, *post hoc* test; stimulus duration, 1.2–2.7 s; adjusted *p* values < 0.05, two-way repeated-measures ANOVA; velocity: *p* = 0.96, two-way repeated-measures ANOVA). This ERT is an orienting-related pupil response and a proxy of the arousal change because of stimulus detection (Wang and Munoz, 2015; Montes-Lourido et al., 2021). In the AS experiment, we also found an increase in pupil size, but dilation was associated with a significant increase in stimulus-induced locomotor activity (pupil: *p* < 0.001, *post hoc* test; stimulus duration, 1–10 s; adjusted *p* values < 0.05, two-way repeated-measures ANOVA; velocity: *p* > 0.01, *post hoc* test; stimulus duration, 3–5.3 s; adjusted *p* values < 0.05; two-way repeated-measures ANOVA; Fig. 2*C*). Finally, we calculated pupil size during baseline periods before sensory stimulation both in the VR and the AS experiments. We found that during locomotion the pupil was significantly larger than in stationary periods both in the AS (*p* < 0.01, paired *t* test; Fig. 2*D*) and in the VR (*p* < 0.01, paired *t* test; Fig. 3*D*) experiments.

These results demonstrate that CNN pupillometry can detect the mouse locomotion-induced and stimulus-evoked pupillary changes and can be used to monitor behavioral state change during head fixation experiments.

### Web browser application to perform real-time pupillometry experiments

**Figure 4.**
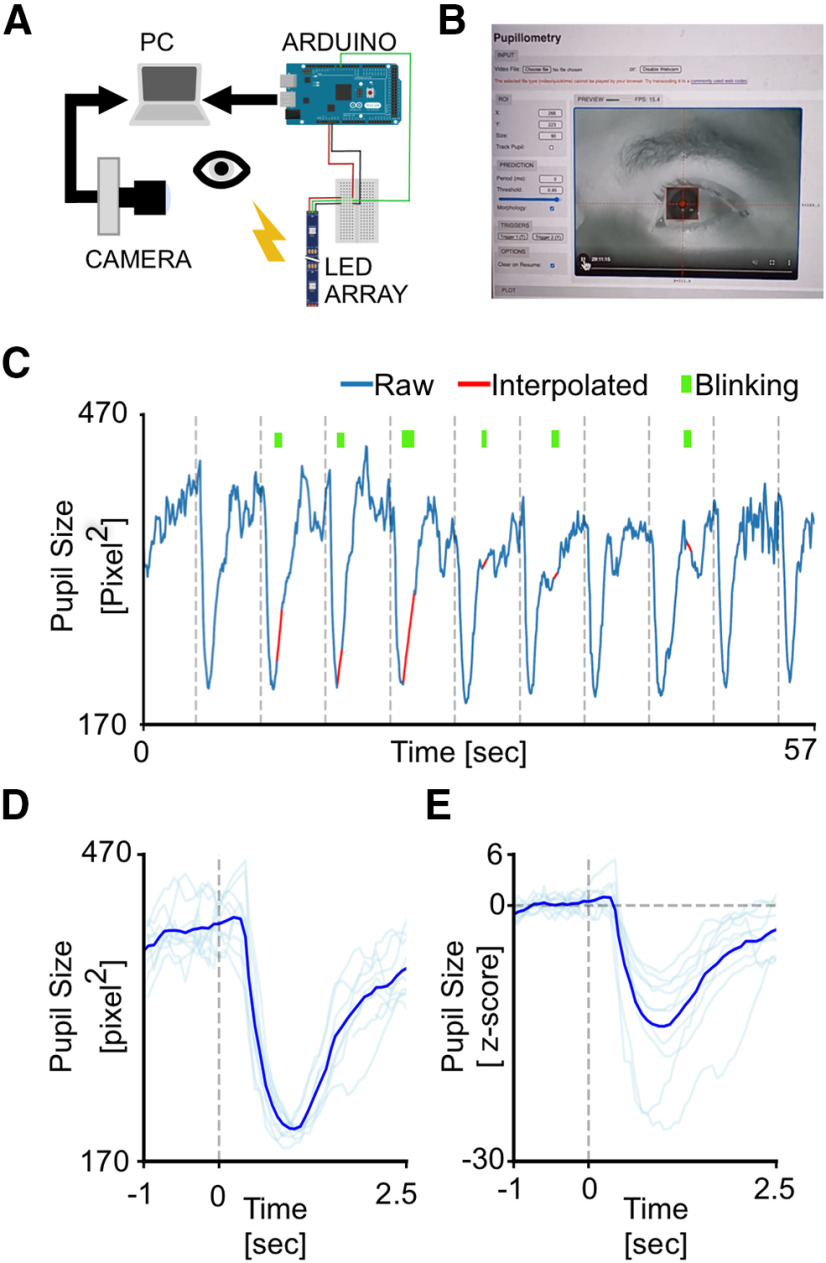
Web browser pupillometry experiment. ***A***, Experimental setup for running the PLR stimulation and in the meantime performing pupillometric recordings. The PC is connected to the Internet by running an instance of the MEYE tool in the web browser. A USB camera, equipped with an IR light source, is focused on the eye of the subject. The photic stimulus is delivered using an LED array driven by an Arduino Due microcontroller board. The Arduino Due board is connected to the PC, emulating a keyboard and sending keystroke stimulus triggers to the MEYE tool. ***B***, A picture of MEYE GUI. The subject during the recording is visualized as a streaming video. An ROI is used to locate the eye, and a preview of the estimation of the pupil is superimposed on the image of the subject. The GUI allows to set different parameters of postprocessing (map thresholding and refinement via mathematical morphology). ***C***, Raw trace of the experiment (blue). Dashed lines locate the onset of flash stimuli. The green rectangles locate the onset and duration of blinks. The samples corresponding to blinks are removed and linearly interpolated (in red). ***D***, Average event-related transient to flash stimulation in raw values. After the onset of the stimulus (dashed line), a strong constriction of the pupil is observed (44.53%). ***E***, The *z*-score of the average event-related transient seen in ***D***. The average nadir amplitude is 14.59 SDs from baseline.

**Figure 5.**
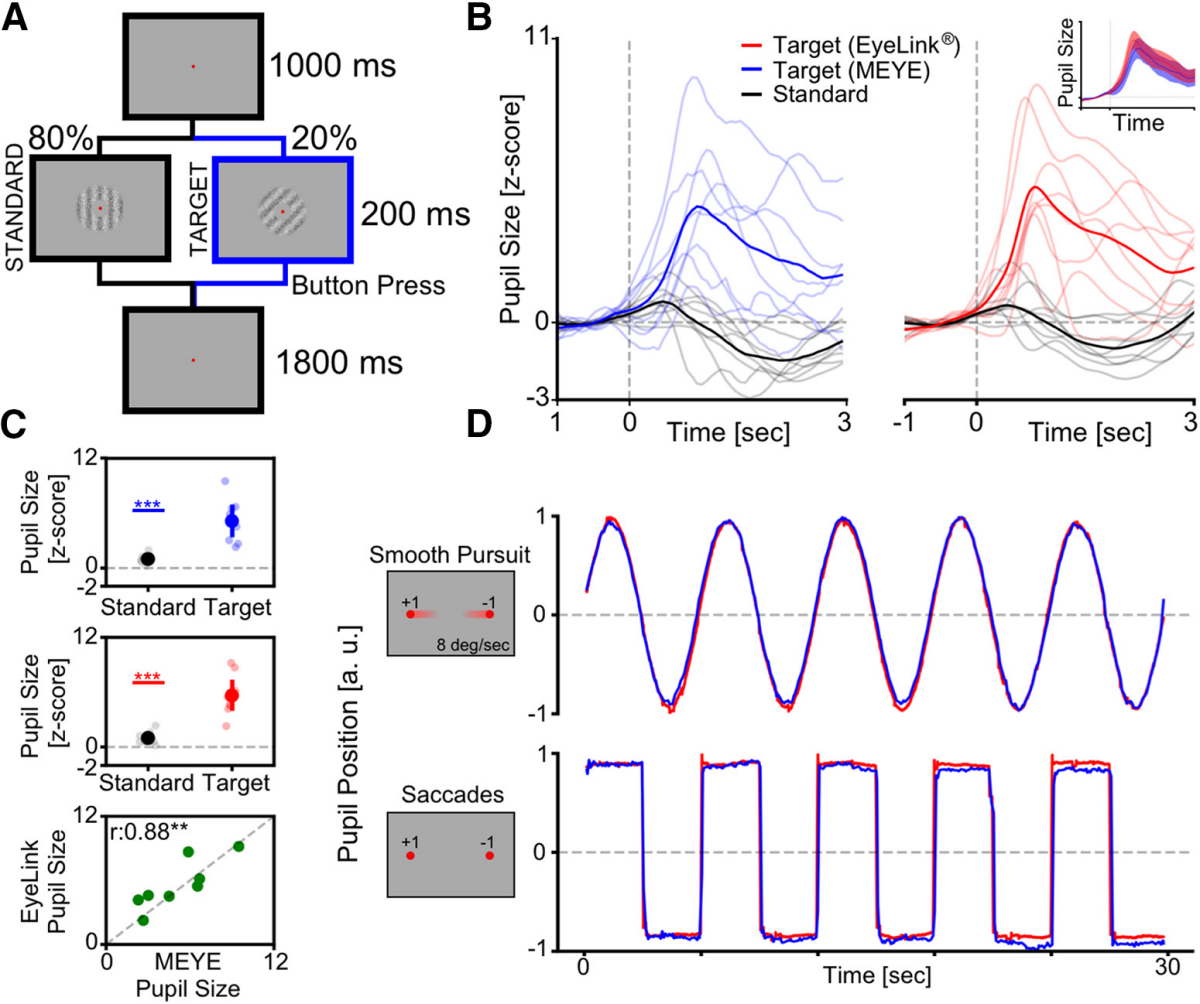
Cognitively driven pupillary changes. ***A***, Visual oddball procedure. The participant is instructed to fixate a small red dot placed at the center of the screen and to push a button only when the Target visual stimulus appears. ***B***, Average pupil waveforms. Average pupil response to Standard and Target stimuli for both the MEYE tool (blue, left) and the EyeLink system (red, right). The inset represents comparison between the evoked response to the Target stimulus in both setups. ***C***, Average pupil response. Difference between the Standard and Target stimuli recording using the MEYE tool (top) and the EyeLink system (middle). The bottom graph represents the correlation between MEYE and EyeLink data. ***D***, Eye movement data. Comparison between the MEYE tool (blue) and EyeLink system (red) during smooth pursuit task (top) and saccades (bottom).

To greatly expand the use of our CNN-based pupillometer, we implemented the CNN in a web browser (MEYE; Fig. 4*B*), and we tested whether it could also be used in humans. To test this possibility, we designed a simple experiment aimed to measure PLR evoked by brief flashes of light on the human eye. The experiment included 10 flash events with an interstimulus interval of 5 s (Fig. 4*C*, dashed vertical lines). The results showed a clear light-induced modulation of pupil size in correspondence with each flash onset. Aligning and averaging all the traces along with the events, PLR can be quantified in both the raw trace (change from baseline, 44.53 ± 0.67%) and *z*-scored trace (SD from baseline, 14.59 ± 2.05; Fig. 3*D*,*E*). To detect whether it is possible to measure cognitively driven pupil signals using the reliable MEYE tool, we performed pupillometry while participants executed an oddball task, a commonly used paradigm for cognitive and attentional measurement. This task is based on the principle by which pupil dilation is stronger in response to rare stimuli and can be used as a physiological marker for the detection of deviant stimuli (Liao et al., 2016). This experiment has been conducted by recording the same eye using both the MEYE tool and an EyeLink 1000 system. According to Google Scholar, the EyeLink 1000 system is one of the most used eye trackers in psychology, psychophysics, and neuroscience, with >17,000 scientific publications mentioning this tool. During the oddball experiment, the subject was instructed to maintain fixation on a small dot presented in the center of the screen, pushing a button only when the Target stimulus appears on the screen and not responding to the Standard stimulus (Fig. 5*A*). Averaging and comparing the responses to Standard and Target gratings result in significantly stronger pupil dilation for the Target stimulus than the Standard stimulus, which is detected by both of the recording systems (MEYE: *p* < 0.001, paired *t* test; EyeLink: *p* < 0.001, paired *t* test; Fig. 5*B*,*C*). No differences have been found for the responses evoked by the Target stimulus between the MEYE tool and the EyeLink system (*p* = 0.327, paired *t* test; Fig. 4*B*, inset). Moreover, the single-subject pupillary evoked amplitudes show a significant positive correlation between the two techniques (ρ = 0.88, *p* = 0.01, Spearman correlation) with >75% of the variability explained by the linear model. Pupil size is known to covary with eye position in video-based measurements (Hayes and Petrov, 2016), producing foreshortening of the pupillary image because the camera is fixed but the eye rotates. To overcome this issue, there are several possible solutions, as follows: the simplest one requires constant fixation throughout each trial, but, if this requirement cannot be satisfied (e.g., in sentence reading), the position of the pupil at each sample can be used to correct and mitigate the estimation error. Thus, we decided to quantify the agreement between positional outputs provided by MEYE and EyeLink for horizontal eye movements. We designed the following two tasks: in the first task, a dot smoothly traveled horizontally on the screen from left to right and vice versa at a velocity of 8°/s and spanning 20°, producing slow and smooth pursuit eye movements. In the other experiment, a dot jumped every 5 s from one position to the other (spanning 20°), producing large, fast, and abrupt saccades. Results (Fig. 4*D*) show that smooth pursuit movements generate a sinusoidal change of position with a good agreement between both systems (MAE, 0.04). The second task, inducing saccades, produces a slightly larger error (MAE, 0.073). This error is mainly because of the much lower sampling rate of MEYE (MEYE, 15 fps; EyeLink, 1000 fps). This means that even if MEYE provides the exact positional information for each sample, it has a lower performance in adequately describing fast eye movements, such as saccades. Thus, MEYE provides the data required for *post hoc* correction of pupil measures, although it should be used with caution for measuring saccades.

### Web browser application to perform pupillometry on videos

**Figure 6.**
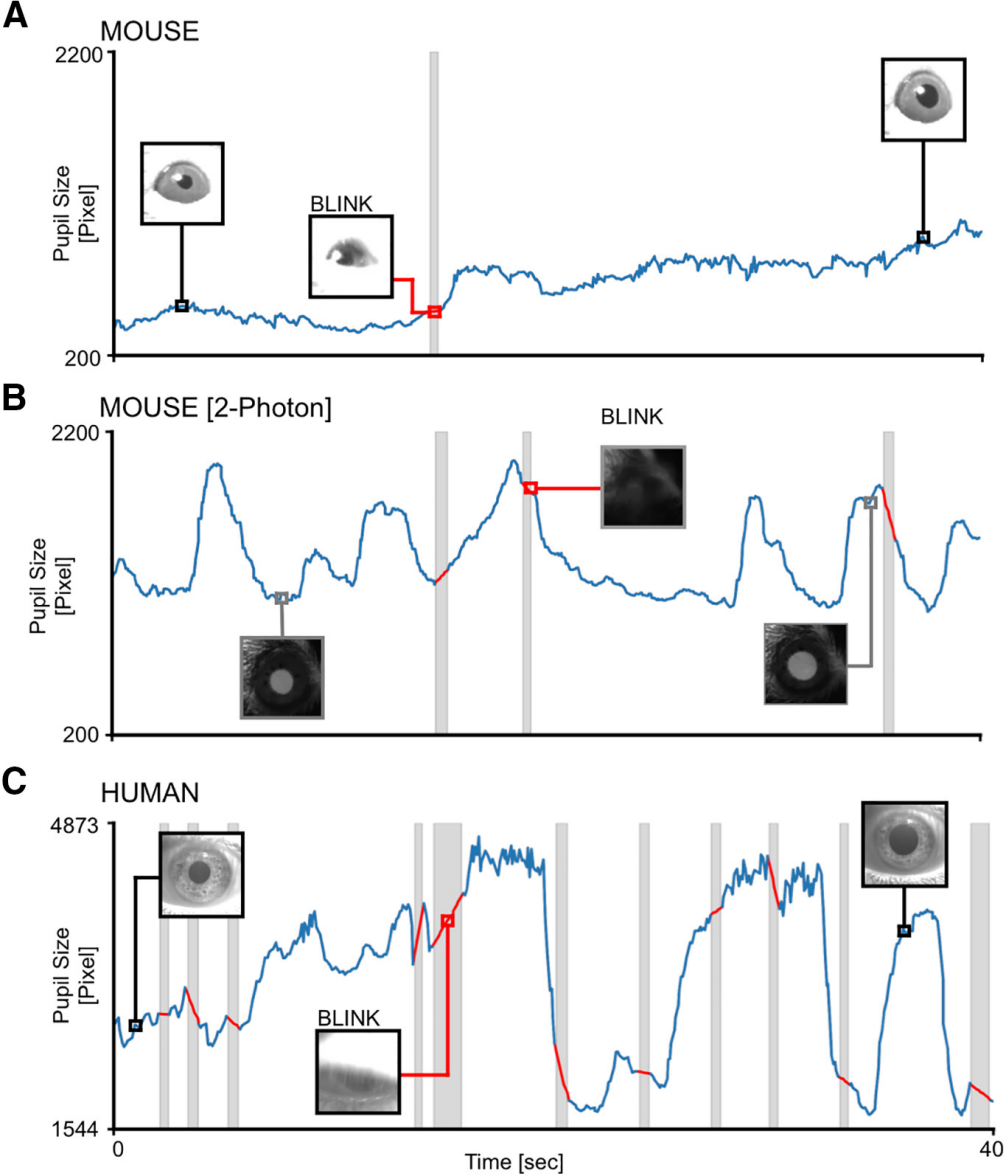
Offline movies analysis. ***A***, Awake head-fixed mouse running on a treadmill, recorded for 40 s. The gray area represents a blink, and the trace of the blink is removed and linearly interpolated (red line). ***B***, Awake mouse during two-photon calcium imaging. Here a brighter pupil is clearly visible with respect to ***A***. Blinking epochs are removed and linearly interpolated. ***C***, Pupillometry performed on a human subject, with a higher blinking rate with respect to mice. In all figures, the inset images represent the ROIs.

MEYE can also be used as an offline tool to analyze pupillometry videos in various file formats, depending on the video codec installed in the web browser. To demonstrate the feasibility of performing pupillometry on videos captured in a variety of situations and in both mice and humans, we sampled three videos with a duration of 40 s from different experiments carried in our laboratory. Each video can be loaded as a demonstration in the web app to reproduce the same plots seen in Figure 6. Three conditions were analyzed. The first condition can be found by pressing the “Mouse” button in the DEMO section of the graphical UI (GUI). It depicts a head-fixed mouse running on a circular treadmill under IR illumination and watching a uniform gray screen at 10 cd/m^2^ (Fig. 6*A*). The second is a mouse under a two-photon microscope (button “2P mouse”), walking on a cylindrical treadmill and showing clear dilation events because of locomotion (Fig. 6*B*). The third is found by pressing the button “Human,” starting 40 s of footage of a human subject wearing VR goggles projecting a uniform gray at 15 cd/m^2^ (Fig. 6*C*). These results show that offline pupillometry can be performed in various conditions and in both mice and humans.

## Discussion

In this work, we demonstrated that MEYE is a sensitive tool that can be used to study pupil dynamics in both humans and mice. Furthermore, by providing eye position, MEYE allows *post hoc* control of the possible effects of eye movements on pupil measures (Hayes and Petrov, 2016). MEYE can detect both locomotion-induced and stimulus-evoked pupil changes with a peak latency of 1 s in a variety of conditions: mice with black pupils in normal illumination conditions; and mice with bright pupils resulting from laser infrared illumination. This flexibility allows the use of MEYE in combination with two-photon, wide-field imaging, and electrophysiological techniques widely adopted in awake or anesthetized mice. Furthermore, MEYE can be used to design stand-alone experiments using cost-effective hardware with performance comparable with that of state-of-the-art commercial software. In this experiment, we used a USB webcam with a varifocal objective that allows focal adjustment concentrated on the eye. The cost of the imaging equipment is <50€ (Tables 2, 3) and requires no knowledge of coding to set up. The flashing stimulus apparatus requires a basic understanding of Arduino boards and can be assembled at a price of <50€. The overall cost of the apparatus is <100€. Our code can be used in two different ways, to satisfy many needs. One way relies on the stand-alone web browser tool, which allows running MEYE on almost any device, from scientific workstations to notebooks or even smartphones. The other way uses a dedicated Python script running the CNN locally on a workstation. This latter case is suited for experiments with specific requirements, like high and stable frame rate or online processing of pupil size in which on-the-fly pupil computer interaction is required.

**Table 2:** Hardware for PLR

Part no.	Description	Quantity	Price (€)	Store	Manufacturer
Walfront5k3psmv97x	USB webcam	1	33.48	Amazon	Walfront
149129	Varifocal M12 lens	1	12.03	Amazon	Sodial
A000062	Microcontroller Arduino Due	1	35	Arduino store	Arduino
1312	4 NeoPixel RGB LEDs	1	6.53	Adafruit	Adafruit
Total amount			87.04		

**Table 3:** Statistical table

Figure	Type of test	Statistical data
Figure 1	No statistical tests	
Figure 2*C*	Two-way repeated-measures ANOVA	Pupil: *F* = 10.56, *p* < 0.001, ng^2^ = 0.43 Velocity: *F* = 1.47, *p* < 0.01, ng^2^ = 0.2
Figure 2*D*	Parametric paired *t* test	*T* = −8.4395, *p* < 0.01, BF_10_ = 14.1
Figure 3*C*	Two-way repeated-measures ANOVA	Pupil: *F* = 7.42, *p* < 0.001, ng^2^ = 0.42 Velocity: *F* = 0.75, *p* < 0.96, ng^2^ = 0.1)
Figure 3*D*	Parametric paired *t* test	*T* = −11.1, *p* < 0.001, BF_10_ = 77.5
Figure 5*C* 1	Parametric paired *t* test	*T* = −4.64, *p* < 0.001, BF_10_ = 62.9
Figure 5*C* 2	Parametric paired *t* test	*T* = −5.41, *p* < 0.001, BF_10_ = 204.03
Figure 5*C* 3	Spearman correlation	*r* = 0.9; 95% CI = [0.46, 0.98], *r*^2^ = 0.78, *p* < 0.01, power = 0.9
Figure 6	No statistical tests	

BF, Bayes factor.

### Comparison with other methods

Valid open-source and commercial alternatives exist, but most of them are dedicated to gazing tracking and/or pupillometry. Commercial options are costly (https://tobii.com, https://sr-research.com, https://neuroptics.com), whereas open-source code requires programming knowledge and most open-source alternatives are explicitly dedicated to one species (Yiu et al., 2019; Privitera et al., 2020). One study (Privitera et al., 2020) assessed pupil dilation in mice through DeepLabCut (Mathis et al., 2018), a technique for 3D markerless pose estimation based on transfer learning. This approach, albeit powerful, is conceptually different since it is trained on user-defined key points instead of on using the entire pupil to perform semantic segmentation. The former technique is more suited to track and locate arbitrary objects on an image, while the latter technique is focused on a more precise quantification of even small changes of the object area since pixelwise segmentation masks are refined iteratively using local and global contexts.

We compared our architecture with the first stage of DeepLabCut, which implements an image segmentation task (leaving out keypoint extraction), which is in common with our pipeline.

For a fair comparison, we trained the image segmentation networks used in DeepLabCut on our dataset. Table 4 shows that our architecture can achieve a higher number of frames per second and a superior segmentation performance (higher dice coefficient) with respect to the DeepLabCut models.

**Table 4:** Comparison among CNN models

CNN model	Train/test set	mDice	FPS (Web*^a^*)	FPS (Keras*^b^*)	FLOPS	Parameters, *n*
mini-UNet	Human/mouse eyes	84.0%	23.2	45.2	0.2G	0.03 M
DeepLabv3+/ResNet-50	ImageNet + finetune on human/mouse eyes	80.1%	<1	28.7	14.1G	26.8 M
DeepLabv3+/Lite-MobileNet- V3-Small	ImageNet + fine-tune on human/mouse eyes	69.0%	18.8	34.8	0.3G	1.1 M

*^a^*Dell Laptop: Intel(R) Core(TM) i7-9750H CPU @ 2.60 GHz; Intel UHD graphics 630 GPU; TensorFlow.js; backend: WebGL Browser: Microsoft edge 90.0.818.56.

*^b^*Ubuntu PC: Intel(R) Core(TM) i9-9900K CPU @ 3.60 GHz; GeForce RTX 2080 Ti GPU; Python 3.6.9 + TensorFlow 2.4.1. FLOPS, FLoating point Operations Per Second; G, gigaFLOPS; M, mefaFLOPS.

### Possible preclinical and clinical applications

The possible contribution of the web app technology resides in its portability: no software needs to be manually installed, and configuration is minimal. Only a clear IR image of the subject’s eye is required. The performances of the tool are dependent on the host computer, but it runs at >10 fps in most of the machines tested. This advantage is particularly useful for settings with limited resources and space or for educational purposes. Web browser-embedded pupillometry will also be crucial for human scientific research, and clinical and preventive medicine. It would also be a promising tool in the recently growing field of telemedicine, given its minimal setup that can run on an average notebook computer or even on a smartphone, and that it allows possible large-scale recruitment of subjects directly in their own homes. This greatly facilitates infant, psychiatric, and motor-impaired patients’ compliance, particularly for longitudinal research designs. We also released an open-source database of eyes composed of >11,000 images in various settings: head-fixed mice (black pupil); head-fixed two-photon imaging mice (white pupil); and human eyes. This dataset will grow over time to introduce new species and new use cases to increase, update, and strengthen MEYE performance. An updated list of planned and executed developments of MEYE can be found in the “Future Development” section of the GitHub Wiki. The possible scenarios can be further expanded in the future, because of the dynamic nature of CNN. It can be updated from the source, providing instantaneous updates on each computer running an instance of the program. Our hope is to create a community that refines and consolidates pupillometric performances to produce a tool that can be applied in different environments.

## Synthesis

Reviewing Editor: Siegrid Löwel, University of Goettingen Decisions are customarily a result of the Reviewing Editor and the peer reviewers coming together and discussing their recommendations until a consensus is reached. When revisions are invited, a fact-based synthesis statement explaining their decision and outlining what is needed to prepare a revision will be listed below. The following reviewer(s) agreed to reveal their identity: Nikolaos Aggelopoulos, Janelle Pakan.

The authors detail a method for pupil diameter analysis and pupil tracking that is open-source, economically implemented and included a user friendly web-based app. The paper is generally well written although there are some semantics that should be addressed to not be misleading in the impact of the proof of principle results, which are not required to be of high impact in this case but simply accurate uses of the applied method. While the developed methods are not specifically novel in their end goal, they seem to perform well in comparison to other expensive commercially available options in humans. An unbiased comparison between their method and the leading open source tool for this type of analysis (DeepLabCut) would seem warranted. A more detailed discussion of the image parameters vital/sufficient/necessary for success are also needed to appeal to the more novice user that such a web-based app is targeted towards and likely most beneficial for.

The manuscript provides open source pupil diameter and tracking analysis tools including a user friendly web app. The advances in the field are not dramatic as methods for pupil analysis are already well established using commercial systems and other open source tools (e.g. DeepLabCut). The performance of the tool is compared to the performance of Eyelink and is found to be comparable but at a lower cost. It is also argued that it is a better tool than DeepLabCut, because it is directly measuring pupil size rather than performing semantic segmentation. The prime advantage of the described tool seems to be the economical methods, ease of user friendly interface, and portability allowing pupillometry also in clinical contexts with simply the use of a mobile phone. While this is not a traditional scientific advancement of the field, it may be quite a useful resource for a more general user without extensive programming experience and/or available resources.

In addition, the authors report an increase in running velocity of mice running on a circular disk during the presentation of an auditory stimulus. While there is not much novelty in observing pupil dilation after auditory stim/locomotion (this has been examined in behaving mice quite extensively, and much more rigorously), claiming novelty of the running speed increases after auditory stimulation would need a much more detailled investigation here. Is this a kind of startle response? We therefore suggest that the authors clarify the semantics of some of their conclusions in this regard. They can then cite the appropriate studies that have also shown similar results directly in the methods for clarity. The authors mention some in the intro, but not exhaustively. Instead of trying to make a novel point here, we would say better to just cite the previous literature and say they could reproduce these effects with their analysis tools. Also N number is quite low to be able to really draw significant new conclusions anyway. In fact, we are not sure if the authors can show the more subtle changes in pupil diameter that come with only arousal and no locomotion in mice with MEYE. They have not shown this directly here. Locomotion induced changes are very dramatic and can generally be detected even with rudimentary image analysis tools (of course the web app interface is still a nice development for the community). Please add a discussion of the importance of this finding for the new tool.

Statistics comments

The statistics involved:

(a) measures of pupil dilation during the presentation of a sound using a permutation test. It is not clear what the permutation test compares. Pupil area at time t1 versus at time t2? Some more detail would be useful.

(b) measures of pupil size with or without locomotion. In this case a t-test was used. It is again insufficiently clear what measurements were compared.

(c) Pearson/Spearman correlations of data from the same dataset analysed with the MEYE method vs using Eyelink. Some more detail would again be useful. While the Methods section refers to the use of the Spearman correlation, the Results section reports a Pearson correlation test used to compare pupil dilation to running speed (e.g. Fig 2C) and further down in comparing pupil dilation measured with MEYE vs as measured with Eylink (lines 305-310).

Software comments:

Brief testing was carried out on pupil data collected in the reviewers labs (from two-photon imaging in mice). The processing was relative fast and the graphical output well presented and informative. At first, the lab’s format (.avi) was not accepted, even though these files are opened by many other programs (e.g. ImageJ, VLC, generic windows viewers etc) - a more flexible/comprehensive input format would make this more useful to a general user, which seems to be the strength of the web app. After convertion to mp4 and loading of the test images, most of the perceptually ‘easier’ data was extracted reasonably well - although not quite as nice as in the manuscript. Perhaps tips on optimizing may be useful in the software or as an easily accessible help link. The more difficult samples generally failed. The reviewer’s lab has had success with these samples through custom pipelines, however, these do inevitably involve extensive manual intervention to fully optimize. The lab did not find the thresholding controls particularly powerful for optimization in the web app. In future versions, these could be expanded and improved upon. Ideally, users could also tweak their images within the app itself - but this could be a future development.

In the lab’s testing, because of the presence of an IR light that reflects in the eye in their set-up, the lab often has a small second circular reflection outside of the pupil range. MEYE often recognized this circular area and included it in the pupil region - often creating a region of ‘two pupils’ or a 8-shaped region of interest. It would seem to us to be trivial to include parameters or implement constraints (even user toggled) that would estimate the max pupil size or to train in which only a single ‘connected’ area should be recognized - there should be no need for two separate regions (unconnected) to be identified.

Finally, it was observed that the tool does not perform well at low contrast situations. The threshold optimization scale could perform better if the image contrast could be adjusted. This might be possible in a pre-processing stage that could be implemented for broader functionality. We suggest that the MEYE tool is improved to offer greater flexibility for image editing and file options, e.g. avi, which is a common format in science labs.

Please clarify whether the tool described is also part of a commercial product, i.e. in addition to the free version, is there a commercial version with greater functionality marketed?

Further comments:

1. Line 189 describes the tone presentation as 10 seconds, however, Fig 2C scale suggests the tone was 20 seconds in duration. Please explain the discrepancy - this is important as it also related to the comment below. Also related, line 267 lists interstimulus interval but not stimulus duration. It would be good to include both here in the results.

2. The pupil changes - particularly in Fig 2C are presented as relatively fast on the presented scale, with 20 seconds of auditory stimulation - which is quite a long stimulation period. Line 262-263 discuss an aim as ‘real-time’ pupil analysis, therefore, evidence of faster pupil fluctuations (i.e. data presented on a more reasonable scale in comparison to velocity changes) would be necessary to appreciate the limits of the ‘real-time’ capacity for pupil monitoring.

3. Lines 274-275: this statement is a bit misleading as a simple untrained stimulus is not quite the same ‘cognitive and emotional processing’ as a trained stimulus-reward association in relation to a behavioural choice paradigm for instance. Please tone down this statement - it is tangential to the point anyway and not required to be convincing of the pupil readout as linked to behavioural state changes (as the authors note in the lines following this). It represent a change in arousal/behavioural state in response to either the sensory stimulation and/or locomotion (this was not specifically separated here in the data presentation (i.e. tone with no locomotion - so one can not tell which) - beyond this is unclear and cognitive/emotional processes can not be inferred with this protocol.

4. Line 278. The use of spontaneous pupil changes here is problematic. Spontaneous changes occur when the animal is stationary and small pupil changes are then correlated to arousal levels. This is in contrast to behavioural state changes (with associated changes in arousal) - including locomotion, that result in larger pupil changes. This is what is reported - responses in relation to locomotion - but these are not spontaneous. If the authors are calling them spontaneous because of the lack of sensory stimulation, this is misleading. Locomotion itself is a stimulus the affects pupil diameter so is not considered spontaneous. If the authors can show small fluctuations in pupil diameter during stationary periods then these would be the ‘spontaneous’ changes in arousal (see details in Vinck et al., 2015, https://pubmed.ncbi.nlm.nih.gov/25892300/).

5. Lines 281-282. As mentioned above, the authors did not separate the locomotion effects from the auditory stimulus presentation. This does not seem to be the point of the study, so is likely not necessary, but the statement that evoked versus state changes can be monitored based on this evidence is misleading. If they want to separate these parameter they need to have sufficient data where the tone was presented and mice where stationary. Otherwise, please revise to be more general in the conclusion and not try to separate arousal/state change due to locomotion from sensory evoked responses.

6. Line 333: presumably this is in reference to Figure 5A-C (not Fig 4). The two-photon mouse data should be elaborated on. Why was this performed, what are the differences and caveats. Fig 5B appears to result in a ‘smoother’ pupil diameter change. This is presumably because of some parameter of the images and not a real change in pupil behaviour. We are happy to see this data included, as there is a large field that many find this useful, but we would suggest the authors devote a paragraph to this analysis and describe remaining potential pitfalls/conditions of use.

7. We appreciate the discussion of DeepLabCut as an alternative analysis method, but a demonstration of this analysis in comparison to MEYE would be very informative. Indeed DeepLabCut requires some additional knowledge and the development of the web app offers a very simple and accessible interface - but one would need to know that MEYE at least approaches the efficiency of DLC (similar to EyeLink). This should be a relatively trivial test to perform.

8. Although the authors undoubtedly want to present their best case scenario data - it would be useful from the user perspective (since the aim is clearly to appeal to a general user) to give examples or a further discussion regarding when MEYE may fail and how to optimize image collection to avoid this.

9. We are missing a clear indication of specific points for future development for the MEYE. For an open-source tool, a discussion of this can be very helpful for the community and lead to greater advancements of mutually beneficial tool development.

10. We are also missing a discussion about the speed of the pupil changes - particularly in relation to neural signals. There are many mentions of the link to cognitive processes, but a small statement with regard to the time scales is warranted - likely in the discussion.

11. The text in Figure 1 appears as low quality at the current figure size - perhaps this is just the PDF, but Figure 2 seems fine. Please check.

12. Line 99: ‘Upsampling and downsampling operations have factor 2’. This is a bit unclear as written. Could the authors either link to Fig. 1C here or rephrase this sentence for clarity.

13. Line 290: likely should be ‘flash’ not ‘flashes’

14. The paragraphs towards the end get very lengthy. May I suggest creating more paragraph breaks/sections for ease of reading.

15. Please expand the section on data analysis. Preferably phrase it in the following way Spearman ρ rank-order (or Pearson?) correlation was performed using the function “x” in pingouin.corr, etc. Please clarify if Spearman or Pearson correlation was used. Please expand on what was being compared using the Z-score, i.e. which data points against which other data points. We believe an ANOVA would have been useful for the time series analyses but perhaps we do not understand what is being compared.

16. With regard to the other text, please use the past tense for the work presented, the equipment used and tests carried out. Reserve the present tense only in describing characteristics of commercial products e.g. line 127 “GPU that can process up to 28 fps” and such. Please do not use the present tense in other contexts. E.g. Line 177: Velocity <b>was</b> monitored ... Line 187: a curved monitor <b>was</b> placed, Line 214: Photic stimulation <b>was</b> delivered ... and so on. There are many other instances where “is” should be replaced with “was”.

17. We are confused by the statement on line 99: ‘Upsampling and downsampling operations have factor 2’. It is probably made worse by the use of the present tense where the past tense should have been used. Perhaps a better phrasing would be that convolutional layer upsampling and downsampling (in the CNN they used) was by a factor of 2. As it is written, it rather gives the impression that all convolution up/downsampling by CNNs is by a factor of 2.

## Author Response

POINT-TO-POINT RESPONSE

The authors detail a method for pupil diameter analysis and pupil tracking that is open-source, economically implemented and included a user friendly web-based app. The paper

is generally well written although there are some semantics that should be addressed to not

be misleading in the impact of the proof of principle results, which are not required to be of

high impact in this case but simply accurate uses of the applied method. While the

developed methods are not specifically novel in their end goal, they seem to perform well in

comparison to other expensive commercially available options in humans.

An unbiased comparison between their method and the leading open source tool for this

type of analysis (DeepLabCut) would seem warranted.

Thank you for raising this point.

We could not perform a direct comparison with DeepLabCut (DLC) in an end-to-end fashion,

as available pre-trained models do not perform well on our multi-specie scenario, and our

dataset is incompatible with and not directly convertible to DLC’s expected label format.

In addition, DLC is trained using user-defined key points and therefore it involves different

steps (respect to the UNET) for the identification of the size and location of an object. Our

11k images dataset is not suitable for direct DLC training, and this means that the two

models should be trained with datasets with different labeling. To overcome this limitation in

performance comparison, we adopted the “DeepLab” CNNs

(https://arxiv.org/pdf/1606.00915.pdf), which are semantic segmentation networks at the

core of DLC. These models are similar to UNET in terms of structure and can be trained

exactly with the same dataset allowing a direct performance comparison. Thus, we trained

and compared different versions of DeepLab with our UNET based pupillometer. We added

a detailed description of the performances of MEYE and DeepLab in the Github Wiki

(https://github.com/fabiocarrara/meye/wiki/MEYE-Models). We found our model to offer a

better trade-off between speed (in FPS) and segmentation performance (in Dice Coefficient).

These data have been included in Table 4.

However, since the MEYE backend model can be dynamically changed (through the

MODELS dropdown menu), we integrated DeepLab into the MEYE webapp. In this way the

user can choose between the UNET (our developed CNN) or the DeepLab (the standard

DLC CNN) models.

A more detailed discussion of the image parameters vital/sufficient/necessary for success

are also needed to appeal to the more novice user that such a web-based app is targeted

towards and likely most beneficial for.

We created a section of the Github Wiki named “Hardware, Image Parameters and Common

Issues", with all the details about tested hardware and acquisition parameters.

https://github.com/fabiocarrara/meye/wiki/Hardware,-Image-Parameters-and-Common Issues

The manuscript provides open source pupil diameter and tracking analysis tools including a

user friendly web app. The advances in the field are not dramatic as methods for pupil

analysis are already well established using commercial systems and other open source tools

(e.g. DeepLabCut). The performance of the tool is compared to the performance of Eyelink

and is found to be comparable but at a lower cost. It is also argued that it is a better tool than

DeepLabCut, because it is directly measuring pupil size rather than performing semantic segmentation.The prime advantage of the described tool seems to be the economical

methods, ease of user friendly interface, and portability allowing pupillometry also in clinical

contexts with simply the use of a mobile phone. While this is not a traditional scientific

advancement of the field, it may be quite a useful resource for a more general user without

extensive programming experience and/or available resources.

In addition, the authors report an increase in running velocity of mice running on a circular

disk during the presentation of an auditory stimulus. While there is not much novelty in

observing pupil dilation after auditory stim/locomotion (this has been examined in behaving

mice quite extensively, and much more rigorously), claiming novelty of the running speed

increases after auditory stimulation would need a much more detailed investigation here.

Is this a kind of startle response?

We therefore suggest that the authors clarify the semantics of some of their conclusions in

this regard. They can then cite the appropriate studies that have also shown similar results

directly in the methods for clarity. The authors mention some in the intro, but not

exhaustively. Instead of trying to make a novel point here, we would say better to just cite

the previous literature and say they could reproduce these effects with their analysis tools.

We definitely agree that our goal was not to discover new effects, but rather use published

ones to test our pupillometry system. The auditory stimulus that we employed is similar to

the conditioned stimulus used in published fear conditioning papers. The saliency of the tone

and the absolute volume can induce defensive sound-induced flight behavior on mice in an

intensity-dependent manner. We eliminated any claim about the novelty of this finding

because other works describing these phenomena exist. For this reason, we added a brief

discussion in the results section and appropriate references explaining the results (line 314-

317).

Papers with auditory stimulus >=20sec:

https://www.ncbi.nlm.nih.gov/pmc/articles/PMC4154579/

https://www.ncbi.nlm.nih.gov/pmc/articles/PMC6596193/

https://www.ncbi.nlm.nih.gov/pmc/articles/PMC5651384/

https://www.ncbi.nlm.nih.gov/pmc/articles/PMC5098193/

https://www.ncbi.nlm.nih.gov/pmc/articles/PMC1142462/

https://www.ncbi.nlm.nih.gov/pmc/articles/PMC6715724/

Also N number is quite low to be able to really draw significant new conclusions anyway. In

fact, we are not sure if the authors can show the more subtle changes in pupil diameter that

come with only arousal and no locomotion in mice with MEYE. They have not shown this

directly here. Locomotion induced changes are very dramatic and can generally be detected

even with rudimentary image analysis tools (of course the web app interface is still a nice

development for the community). Please add a discussion of the importance of this finding

for the new tool.

Thank you for prompting us to test this possible limitation of the tool. We added a new figure

with an entire new experiment employing visual stimulation (N=5). In this experiment, we

show an event-related transient of the pupil in absence of significant locomotor activity. This

demonstrates that the tool is able to detect subtle fluctuation in arousal (orienting response)

given by stimulus detection in mice (line 321-324).Statistics comments

The statistics involved:

(a) measures of pupil dilation during the presentation of a sound using a permutation test. It

is not clear what the permutation test compares. Pupil area at time t1 versus at time t2?

Some more detail would be useful.

We changed the permutation test used here with a two-way repeated-measures ANOVA as

suggested in comment 15 and added the requested details in the manuscript methods

section (line 221-227).

(b) measures of pupil size with or without locomotion. In this case a t-test was used. It is

again insufficiently clear what measurements were compared.

We measured the average pupil size during stationary or moving periods. Moving periods

are defined as the animal moving at a velocity >=10% of its maximal speed during the entire

session. We added this detail in the manuscript methods (line 228-230).

(c) Pearson/Spearman correlations of data from the same dataset analysed with the MEYE

method vs using Eyelink. Some more detail would again be useful. While the Methods

section refers to the use of the Spearman correlation, the Results section reports a Pearson

correlation test used to compare pupil dilation to running speed (e.g. Fig 2C) and further

down in comparing pupil dilation measured with MEYE vs as measured with Eylink (lines

305-310).

We addressed this problem performing Spearman correlation in all the cases as stated in the

methods section. We also expanded the method section related to this aspect (line 283-

286).

Software comments:

Brief testing was carried out on pupil data collected in the reviewers labs (from two-photon

imaging in mice). The processing was relatively fast and the graphical output well presented

and informative. At first, the lab’s format (.avi) was not accepted, even though these files are

opened by many other programs (e.g. ImageJ, VLC, generic windows viewers etc) - a more

flexible/comprehensive input format would make this more useful to a general user, which

seems to be the strength of the web app.

We are really pleased to hear that reviewers tried MEYE, thank you very much.

We agree that support for the ‘.avi’ files could improve the usability of the web app to a

general user (we also use the .avi format for 2P experiments) but unfortunately such a

feature it’s impossible for now to implement due to HTML5 development policies specifically

not supporting video playback for the .avi format. We addressed this problem in two ways:

first, we provided an extensively commented python notebook for offline analysis and

postprocessing of .avi videos. Second, we added a link on the GUI to an online avi to MP4

converter. We hope that in the future HTML5 could also support .avi video playback,

however, many other high quality video formats are supported by HTML5, see

https://en.wikipedia.org/wiki/HTML5_video for a list of all the supported file formats.

We added a discussion about this limitation in the section ‘Hardware, Image Parameters and

Common Issues’ in the Github Wiki

After convertion to mp4 and loading of the test images, most of the perceptually ‘easier’ data

was extracted reasonably well - although not quite as nice as in the manuscript. Perhaps tips

on optimizing may be useful in the software or as an easily accessible help link. The more difficult samples generally failed. The reviewer’s lab has had success with these samples

through custom pipelines, however, these do inevitably involve extensive manual

intervention to fully optimize.

We improved the image optimization in three different ways: first, we introduced in the Web

app a PREPROCESSING section, a series of image enhancement tools to better control

image parameters. The user can change these parameters and easily find the best condition

to achieve correct pupil size measurement. Please note that in our hands another possible

solution for difficult samples of 2P experiments is to invert image colors, a manipulation that

now is available in the GUI as a checkbox. Second, In the LINKS section of the MEYE GUI

we created a link to the manual section called “Hardware, Image Parameters and common

issues", in which the best hardware setup and image optimization are explained in detail

(https://github.com/fabiocarrara/meye/wiki/Hardware,-Image-Parameters-and-Common Issues). Finally, we introduced a new selectable model based on DeepLab CNN that can be

used interchangeably with the original UNET CNN.

The lab did not find the thresholding controls particularly powerful for optimization in the web

app. In future versions, these could be expanded and improved upon. Ideally, users could

also tweak their images within the app itself - but this could be a future development.

As stated in the previous point, we added new image preprocessing tools in the GUI: image

color inversion, brightness, gamma, and contrast controls. Moreover, we plan to add

customizable mathematical morphology as post processing tools.

In the lab’s testing, because of the presence of an IR light that reflects in the eye in their setup, the lab often has a small second circular reflection outside of the pupil range. MEYE

often recognized this circular area and included it in the pupil region - often creating a region

of ‘two pupils’ or a 8-shaped region of interest. It would seem to us to be trivial to include

parameters or implement constraints (even user toggled) that would estimate the max pupil

size or to train in which only a single ‘connected’ area should be recognized - there should

be no need for two separate regions (unconnected) to be identified.

Thanks for pointing this out. To solve this problem we introduced in MEYE GUI the

Morphology option (available in the PREDICTION section). This box should be checked to

exclude possibly identified secondary areas. The exclusion is based on size, only the biggest

area is retained. We would like to add an entire set of morphology tools that can be manually

toggled to improve pupil detection, even if our goal is to improve the model until we reach a

level of optimal performance in each situation.

Finally, it was observed that the tool does not perform well at low contrast situations. The

threshold optimization scale could perform better if the image contrast could be adjusted.

This might be possible in a pre-processing stage that could be implemented for broader

functionality. We suggest that the MEYE tool is improved to offer greater flexibility for image

editing and file options, e.g. avi, which is a common format in science labs.

We added brightness and contrast controls in the app, with the corresponding descriptions in

the Github Wiki.

Please clarify whether the tool described is also part of a commercial product, i.e. in addition

to the free version, is there a commercial version with greater functionality marketed?

The code is provided with the General Public License version 3 (GPLv3). It is a copyleft license granting open-source permissions for users including the right to download, make

changes, redistribute and modify copies of the software. Moreover, GPLv3 states that

modified versions of the program require making the changed source code available to

users, under the GPLv3 terms.

Further comments:

1. Line 189 describes the tone presentation as 10 seconds, however, Fig 2C scale suggests

the tone was 20 seconds in duration. Please explain the discrepancy - this is important as it

also related to the comment below. Also related, line 267 lists interstimulus interval but not

stimulus duration. It would be good to include both here in the results.

We corrected the typo in the text and added the requested details.

2. The pupil changes - particularly in Fig 2C are presented as relatively fast on the presented

scale, with 20 seconds of auditory stimulation - which is quite a long stimulation period. Line

262-263 discuss an aim as ‘real-time’ pupil analysis, therefore, evidence of faster pupil

fluctuations (i.e. data presented on a more reasonable scale in comparison to velocity

changes) would be necessary to appreciate the limits of the ‘real-time’ capacity for pupil

monitoring.

We adjusted the time scale of Fig 2C plots to 15 sec (5 pre and 10 post-stimulus) time

window and reduced median filtering to better appreciate faster pupil and locomotor

fluctuations.

3. Lines 274-275: this statement is a bit misleading as a simple untrained stimulus is not

quite the same ‘cognitive and emotional processing’ as a trained stimulus-reward association

in relation to a behavioural choice paradigm for instance. Please tone down this statement -

it is tangential to the point anyway and not required to be convincing of the pupil readout as

linked to behavioural state changes (as the authors note in the lines following this). It

represent a change in arousal/behavioural state in response to either the sensory stimulation

and/or locomotion (this was not specifically separated here in the data presentation (i.e. tone

with no locomotion - so one can not tell which) - beyond this is unclear and

cognitive/emotional processes can not be inferred with this protocol.

We modified the sentence accordingly. In addition we changed the article referenced here,

from an article involving rewarded stimuli to papers involving orienting response pupil-related

dilations.

4. Line 278. The use of spontaneous pupil changes here is problematic. Spontaneous

changes occur when the animal is stationary and small pupil changes are then correlated to

arousal levels. This is in contrast to behavioural state changes (with associated changes in

arousal) - including locomotion, that result in larger pupil changes. This is what is reported -

responses in relation to locomotion - but these are not spontaneous. If the authors are

calling them spontaneous because of the lack of sensory stimulation, this is misleading.

Locomotion itself is a stimulus the affects pupil diameter so is not considered spontaneous. If

the authors can show small fluctuations in pupil diameter during stationary periods then

these would be the ‘spontaneous’ changes in arousal (see details in Vinck et al., 2015,

https://pubmed.ncbi.nlm.nih.gov/25892300/).

We agree that it is very important to test our system also with smaller pupil changes typical

of pupil modulation during stationary periods. Therefore, although our previous data showed

that in humans MEYE was capable of detecting pupil fluctuation induced by an oddball paradigm in the absence of movement, we decided to test this possibility also in the mouse.

In Figure 3 we show the results of an experiment in which the mouse navigates a virtual

reality corridor for 5 minutes. After this period a squared visual stimulus was presented in the

binocular portion of the visual field. We detected a significant pupillary dilation after the onset

of the visual stimulus, and no changes in evoked locomotion, suggesting that our system can

capture subtle pupil size modulation unrelated to locomotion. Please note that to avoid

confusion in the terminology we eliminated any reference to spontaneous pupil changes.

5. Lines 281-282. As mentioned above, the authors did not separate the locomotion effects

from the auditory stimulus presentation. This does not seem to be the point of the study, so

is likely not necessary, but the statement that evoked versus state changes can be

monitored based on this evidence is misleading. If they want to separate these parameter

they need to have sufficient data where the tone was presented and mice where stationary.

Otherwise, please revise to be more general in the conclusion and not try to separate

arousal/state change due to locomotion from sensory evoked responses.

The referee is correct that one possibility to see the sensitivity of MEYE for pupil fluctuations

unrelated to locomotion is to separately evaluate pupil size in stationary trials. However, the

number of trials in the auditory experiment was not sufficient to have enough stationary

trials. However, as described in the previous point we have performed another experiment

using visual cues that do not elicit stimulus-induced locomotion. This experiment clearly

shows that our system is able to reveal stimulus evoked pupillary dilation in absence of

significant stimulus induced increment of velocity.

6. Line 333: presumably this is in reference to Figure 5A-C (not Fig 4). The two-photon

mouse data should be elaborated on. Why was this performed, what are the differences and

caveats. Fig 5B appears to result in a ‘smoother’ pupil diameter change. This is presumably

because of some parameter of the images and not a real change in pupil behaviour. We are

happy to see this data included, as there is a large field that many find this useful, but we

would suggest the authors devote a paragraph to this analysis and describe remaining

potential pitfalls/conditions of use.

MEYE can also be used as an offline tool to analyze pupillometry videos in various file

formats, depending on the video codec installed in the web browser. To demonstrate the

feasibility to perform pupillometry on videos captured in a variety of situations in both mice

and humans, we provide examples of pupil size quantifications in fig.5 (now fig.6). The B

panel depicts a 40 seconds pupil trace of a mouse in a 2-photon microscopy experiment

while recording visual cortical activity. Concerning the “smoothness” of the B panel trace,

this could have been partly due to the different y-scale used. In the new figure (fig. 6) we

now report fA and B panels with the same scales on the plot axes. Differences between the

traces could also be due to the fact that each video was recorded using different cameras

and setups (different luminance level, IR illumination, image resolution, type of head fixation

and treadmill used), and with different habituation of the mouse to the setup, making the

comparison between the different conditions hard to do. The videos were sampled from

different experiments with no sensory stimulation, but locomotion and other movements

could be present. For instance, we noticed that the eye of the animal in the 2P recording

(panel B) was less mobile than in the condition of panel A, possibly contributing to the

smoothness of the trace.

7. We appreciate the discussion of DeepLabCut as an alternative analysis method, but a demonstration of this analysis in comparison to MEYE would be very informative. Indeed

DeepLabCut requires some additional knowledge and the development of the web app

offers a very simple and accessible interface - but one would need to know that MEYE at

least approaches the efficiency of DLC (similar to EyeLink). This should be a relatively trivial

test to perform.

We could not perform a direct comparison with DeepLabCut (DLC) in an end-to-end fashion,

as available pretrained models do not perform well as-is on our multi-specie scenario, and

our dataset is incompatible with and not directly convertible to DLC’s expected label format.

In addition, DLC is trained using user-defined key points and therefore it involves different

steps (respect to the UNET) for the identification of the size and location of an object. Our

11k images dataset is not suitable for direct DLC training, and this means that the two

models should be trained with datasets with different labeling. To overcome this limitation in

performance comparison, we adopted the “DeepLab” CNNs

(https://arxiv.org/pdf/1606.00915.pdf), which are semantic segmentation networks at the

core of DLC. These models are similar to UNET in terms of structure and can be trained

exactly with the same dataset allowing a direct performance comparison. Thus, we trained

and compared different versions of DeepLab with our UNET based pupillometer. We added

a detailed description of the performances of MEYE and DLC in the Github Wiki

(https://github.com/fabiocarrara/meye/wiki/MEYE-Models). We found our model to offer a

better trade-off between speed (in FPS) and segmentation performance (in Dice Coefficient).

These data have been included in Table 4 (line:435-439).

However, since the MEYE backend model can be dynamically changed (through the

MODELS dropdown menu), we integrated DeepLab into the MEYE web app. In this way, the

user can choose between the UNET (our developed CNN) or the DeepLab (the standard

DLC CNN) models.

8. Although the authors undoubtedly want to present their best case scenario data - it would

be useful from the user perspective (since the aim is clearly to appeal to a general user) to

give examples or a further discussion regarding when MEYE may fail and how to optimize

image collection to avoid this.

We added in the Github Wiki a section describing the best practice to avoid common issues

and pitfalls, moreover, we added to the web app the possibility to preprocess the image

parameters to increase the flexibility of MEYE. We also underscored that IR light is

necessary for accurate pupil measurements. Another point that is worth mentioning here is

that the CNN implemented in a web app is capable of learning. Therefore, MEYE can be

updated over time by adding new examples in the training dataset in which the actual model

fails. We plan to perform periodic updates of the model that are able to refine and improve

the actual performances of the CNN over time.

9. We are missing a clear indication of specific points for future development for the MEYE.

For an open-source tool, a discussion of this can be very helpful for the community and lead

to greater advancements of mutually beneficial tool development.

Thank you for this comment. We added a link in the Discussion to a page in the Github Wiki

titled “Future Developments” in which we delineate our main future perspective.

10. We are also missing a discussion about the speed of the pupil changes - particularly in

relation to neural signals. There are many mentions of the link to cognitive processes, but a

small statement with regard to the time scales is warranted - likely in the discussion.We added a statement regarding time scales in the discussion (line 405-406).

11. The text in Figure 1 appears as low quality at the current figure size - perhaps this is just

the PDF, but Figure 2 seems fine. Please check.

We uploaded an image at a higher resolution.

12. Line 99: ‘Upsampling and downsampling operations have factor 2’. This is a bit unclear

as written. Could the authors either link to Fig. 1C here or rephrase this sentence for clarity.

We rephrased the sentence.

13. Line 290: likely should be ‘flash’ not ‘flashes’

The typo has been corrected.

14. The paragraphs towards the end get very lengthy. May I suggest creating more

paragraph breaks/sections for ease of reading.

We split the discussion into three paragraphs.

15. Please expand the section on data analysis. Preferably phrase it in the following way

Spearman ρ rank-order (or Pearson?) correlation was performed using the function “x” in

pingouin.corr, etc. Please clarify if Spearman or Pearson correlation was used. Please

expand on what was being compared using the Z-score, i.e. which data points against which

other data points. We believe an ANOVA would have been useful for the time series

analyses but perhaps we do not understand what is being compared.

We clarified in the text that we employed only Spearman ρ rank-order correlation.

We also added a description of what comparison we carried out using Z-score.

As suggested, we changed the permutation tests with two-way repeated-measures ANOVA

in the time series analysis.

16. With regard to the other text, please use the past tense for the work presented, the

equipment used and tests carried out. Reserve the present tense only in describing

characteristics of commercial products e.g. line 127 “GPU that can process up to 28 fps” and

such. Please do not use the present tense in other contexts. E.g. Line 177: Velocity was

monitored ... Line 187: a curved monitor was placed, Line 214: Photic stimulation was

delivered ... and so on. There are many other instances where “is” should be replaced with

"was”.

We corrected the errors.

17. We are confused by the statement on line 99: ’Upsampling and downsampling

operations have factor 2’. It is probably made worse by the use of the present tense where

the past tense should have been used. Perhaps a better phrasing would be that

convolutional layer upsampling and downsampling (in the CNN they used) was by a factor of

2. As it is written, it rather gives the impression that all convolution up/downsampling by

CNNs is by a factor of 2.

We rephrased the sentence
