# Erratum: Mazziotti et al., “MEYE: Web App for Translational and Real-Time Pupillometry”

**DOI:** 10.1523/ENEURO.0122-22.2022

**Published:** 2022-04-08

**Authors:** Raffaele Mazziotti, Fabio Carrara, Aurelia Viglione, Leonardo Lupori, Luca Lo Verde, Alessandro Benedetto, Giulia Ricci, Giulia Sagona, Giuseppe Amato, Tommaso Pizzorusso

**Affiliations:** 1 Department of Neuroscience, Psychology, Drug Research and Child Health (NEUROFARBA), University of Florence, 50135 Florence, Italy; 2 Institute of Neuroscience, National Research Council, 1-56124 Pisa, Italy; 3 Department of Developmental Neuroscience, IRCCS Stella Maris Foundation, 56128 Pisa, Italy; 4 Laboratory of Biology BIO@SNS, Scuola Normale Superiore, 1-56124 Pisa, Italy; 5 Istituto di Scienza e Tecnologia dell’Informazione (ISTI), 1-56124 Pisa, Italy; 6 Department of Translational Research on New Technologies in Medicine and Surgery, University of Pisa, 56127 Pisa, Italy

In the article “MEYE: Web App for Translational and Real-Time Pupillometry,” by Raffaele Mazziotti, Fabio Carrara, Aurelia Viglione, Leonardo Lupori, Luca Lo Verde, Alessandro Benedetto, Giulia Ricci, Giulia Sagona, Giuseppe Amato, and Tommaso Pizzorusso, which published online on September 13, 2021, Leonardo Lupori’s affiliation was listed incorrectly. Leonardo Lupori’s affiliation is Department of Developmental Neuroscience, IRCCS Stella Maris Foundation, 56128 Pisa, Italy. The article has been corrected online, and the correct author affiliations appear below.

